# Biomechanical effects of individualized artificial titanium alloy lamina implantation after laminectomy: A finite element analysis

**DOI:** 10.3389/fbioe.2022.1019510

**Published:** 2022-11-17

**Authors:** Xuqiang Gong, Aobo Zhang, Qing Han, Yang Wang, Yang Liu, Jianhang Jiao, Jing Yue, Hao Chen, Wangwang Luo, Jincheng Wang, Minfei Wu

**Affiliations:** Department of Orthopedics, The Second Hospital of Jilin University, Changchun, China

**Keywords:** laminectomy, artificial lamina, finite element analysis, posterior ligamentous complex, lumbar decompression

## Abstract

**Background and objectives:** Laminectomy is a common surgical procedure in spine surgery. However, disruption of the posterior ligamentous complex of the spine may lead to a range of postoperative complications. Artificial lamina as a kind of bionic implant can well restore the posterior spinal structure. In this study, an individualized artificial titanium alloy lamina was designed to reconstruct the posterior spinal structure after laminectomy and explored its biomechanical effects, which could provide a theoretical basis for the clinical application of the artificial lamina.

**Methods**: Three finite element models were constructed, namely the nonlinear and non-homogeneous intact model of the whole lumbar spine, the lumbar decompression alone surgical model, and the artificial lamina implantation surgical model. The range of motion, intradiscal pressure, and annulus fibrosus peak stress were compared between the three models at the surgical and adjacent segments. The stresses of the artificial lamina and fixation screws were also analyzed for the four movement states.

**Results**: Compared with the intact model, the lumbar decompression alone surgical model showed an increase in range of motion, intradiscal pressure, and annulus fibrosus peak stresses at the surgical segment and adjacent segments under all conditions. The artificial lamina implantation surgical model showed an increase in these measurements only in flexion, increasing by 7.5%–22.5%, 7.6%–17.9%, and 6.4%–19.3%, respectively, over the intact model, while there was little difference under other conditions. The peak stresses in both the screw and the artificial lamina were highest in axial rotation, i. e. 46.53 MPa and 53.84 MPa, respectively. Screw stresses were concentrated on the connection between the screw and the artificial lamina, and artificial lamina stresses were concentrated on the spinous root, around the screw hole, and the contact with the vertebral body.

**Conclusion**: An individualized artificial titanium alloy lamina can effectively reduce the range of motion, intradiscal pressure, and annulus fibrosus stress at the surgical segment and adjacent segments. The application of artificial lamina could better preserve the biomechanical properties of the intact lumbar spine and reduce the risk of adjacent segmental disease.

## Introduction

Posterior lumbar laminectomy alone is the traditional surgical method for the treatment of symptomatic lumbar spinal stenosis and intraspinal tumors, which allows sufficient decompression and tumor resection by removing the posterior spinal structures (Hamawandi et al., 2019; Wang et al., 2020). However, laminectomy might cause epidural fibrous tissue proliferation in the lamina defect area, thereby developing new compression and increasing the difficulty of revision surgery (Avellanal et al., 2014). More importantly, it is well documented that laminectomy can lead to changes in the biomechanics of the lumbar spine, resulting in lumbar instability (Kim et al., 2015; Veisari and Haghpanahi, 2021). All of these factors may result in the development of failed back surgery syndrome (FBSS) after lumbar spine surgery (Sebaaly et al., 2018).

A large number of researchers have dedicated themselves to developing a vertebral plate replacement to restore the posterior spinal structure to prevent FBSS, and the artificial lamina, as a bionic implant, is well suited to achieve this purpose (Li et al., 2020). Nevertheless, there are few studies on the artificial lamina, especially in biomechanics. Liu et al. (2021) designed an individualized artificial lamina of PEEK material and performed biomechanical analysis to demonstrate the role of the artificial lamina in reconstructing the posterior structure of the lumbar spine and reducing the incidence of postoperative complications in the lumbar spine. However, the PEEK is a biologically inert material that is not conducive to osseointegration (Ma and Tang, 2014). On the contrary, titanium alloy materials have good biocompatibility and are currently the best bio metallic materials and the material of choice for bone implants (Lv et al., 2015; Jing et al., 2020). While to the best of our knowledge, biomechanical analysis of individualized artificial titanium alloy lamina has not been reported.

Therefore, an individualized artificial titanium alloy lamina was designed and biomechanical analysis was performed using the finite element method in this study. Finite element analysis (FEA) can realistically simulate the surgical procedure and the biomechanical effects of the prosthesis on the body (Finley et al., 2018). A three-dimensional nonlinear inhomogeneous finite element model of L1-L5 was developed and two surgical procedures were simulated, including lumbar laminectomy decompression alone (DA) and laminectomy followed by artificial lamina implantation (ALI). By evaluating the biomechanical differences between the three models, we explored the biomechanical properties of the artificial titanium alloy lamina implanted after laminectomy and expected to provide a theoretical basis for further clinical applications of the artificial lamina.

## Materials and methods

### FE modeling of the lumbar spine

**FIGURE 1:**
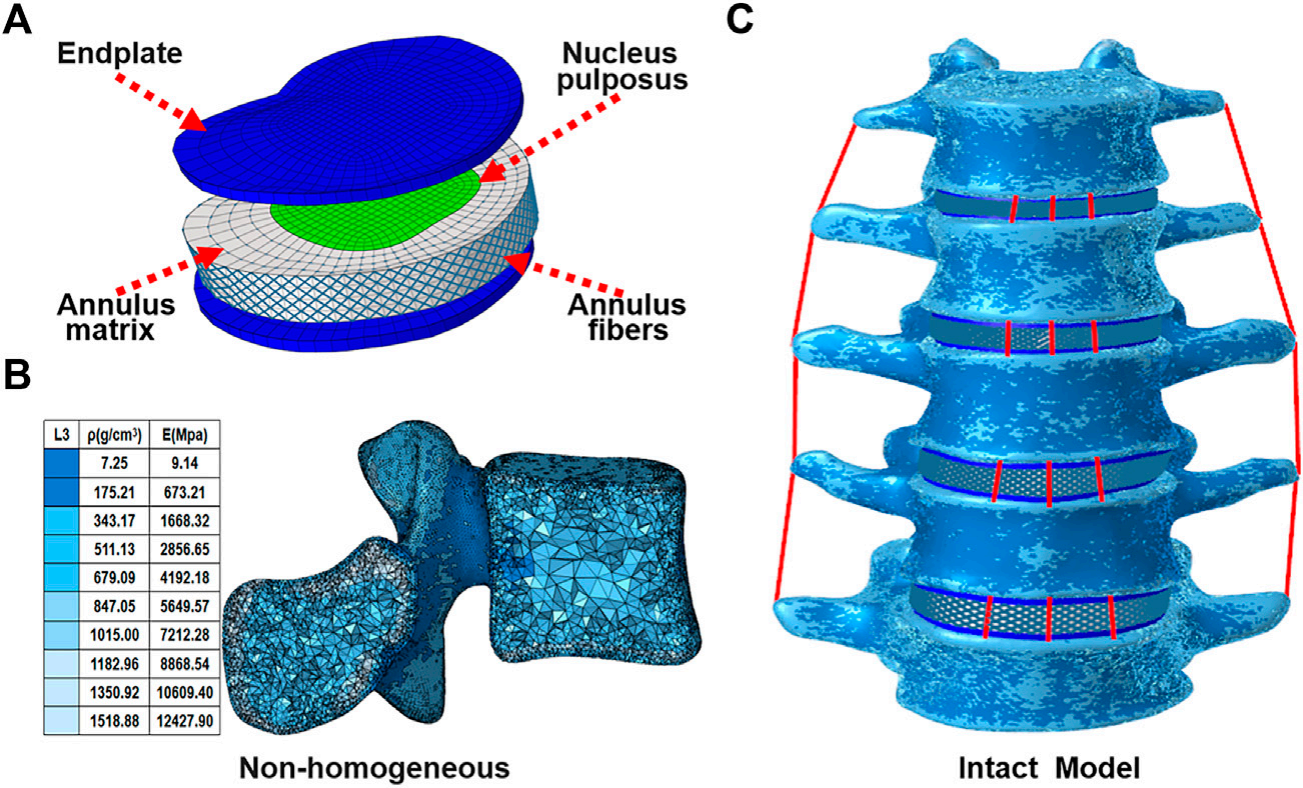
FE model of the lumbar spine:**(A)** Disc and endplate,**(B)** Vertebral body,**(C)** L1-L5.

A three-dimensional nonlinear inhomogeneous FE model of L1-L5 was created based on computed tomography (CT) images of a healthy young male (Figure 1C). The CT images of a participant were obtained at intervals of 0.625 mm (Dual Source CT; Siemens, Munich, Germany). Mimics 21.0 (Materialise, Inc., Leuven, Belgium) was provided to reconstruct the geometric structure of the L1–L5. Initial smoothing and model repair with 3-Matic 13.0 (Materialise, Inc., Leuven, Belgium) and creation of discs, endplates, and facet joints (Figure 1A). These models were then imported into Hypermesh 16.0 (Altair Engineering, Troy, Michigan, United States) for meshing. The vertebral body was divided into a tetrahedral mesh. The intervertebral disc, endplates, and facet joints were divided into a hexahedral mesh. The thickness of the endplate is 0.8 mm, and the thickness of the facet joints is 0.2 mm (Kim et al., 2014). The vertebral body was assigned non-homogeneous values based on CT gray values in Mimics 21.0 software (Materialize, Leuven, Belgium) (Figure 1B) (Rho et al., 1995; Rana et al., 2020). Both the fluid-like behavior of the nucleus pulposus and the hyperelastic properties of the annulus matrix were modeled using the isotropic, incompressible, hyperelastic Mooney-Rivlin formulation (Schmidt et al., 2007). Annulus fibers consist of tension-only truss elements embedded into the annulus matrix, and the fiber stiffness gradually increases from inside to outside (Kim et al., 2014). The anterior longitudinal ligament, posterior longitudinal ligament, intertransverse ligament, ligamentum flavum, capsular ligament, interspinous ligament, and supraspinous ligament were modeled by using tension-only truss elements (Kim et al., 2014; Guo and Fan, 2018; Rana et al., 2020). The files of all models were imported into Abaqus (version 6.14; SIMULIA Inc.) in inp format. Facet joints were approximated by frictionless contact surfaces, and other contact surfaces are defined as Tie (Kim et al., 2014). Finally, nonlinear analysis is carried out in FEA software Abaqus (version 6.14; SIMULIA Inc.). Material properties of tissues of the lumbar spine model were extracted from the literature (Table1).

**TABLE 1:** Material properties of the model.

Component	Young modulus (MPa)	Cross-section (mm^2^)	Poisson’s ratio	Element type	References
Vertebrae (L1-L5)	ρ=47+1.112*HU		0.3	C3D4	Rho et al. (1995); Rana et al. (2020)
	$E=0.63\rho^{1.35}$				
Disc					
Nucleus pulposus	Mooney-Rivlin: c10 = 0.12, c01 = 0.03			C3D8	Schmidt et al. (2007)
Annulus matrix	Mooney-Rivlin: c10 = 0.18, c01 = 0.045			C3D8	Schmidt et al. (2007)
Annulus fibers	360–550		0.3	T3D2	Kim et al. (2014)
Endplate	4,000		0.3	C3D8	Kim et al. (2014)
Facet	23.8		0.4	C3D8	Kim et al. (2014)
Ligament					Kim et al. (2014); Rana et al. (2020)
Anterior longitudinal	7.8 (<12%)	63.7		T3D2	
	20 (>12%)				
Posterior longitudinal	10 (<11%)	20		T3D2	
	20 (>11%)				
Ligamentum flavum	15 (<6.2%)	40		T3D2	
	19.5 (>6.2%)				
Capsular	7.5 (<25%)	30		T3D2	
	32.9 (>25%)				
Interspinous	10 (<14%)	40		T3D2	
	11.6 (>14%)				
Supraspinous	8.0 (<20%)	30		T3D2	
	15 (>20%)				
Intertransverse	10 (<18%)	1.8		T3D2	
	58.7 (>18%)				
Implants					Kim et al. (2014)
Artificial lamina, screws (Ti6Al4V)	110,000		0.3	C3D4	

### Design of an individualized artificial titanium alloy lamina

**FIGURE 2:**
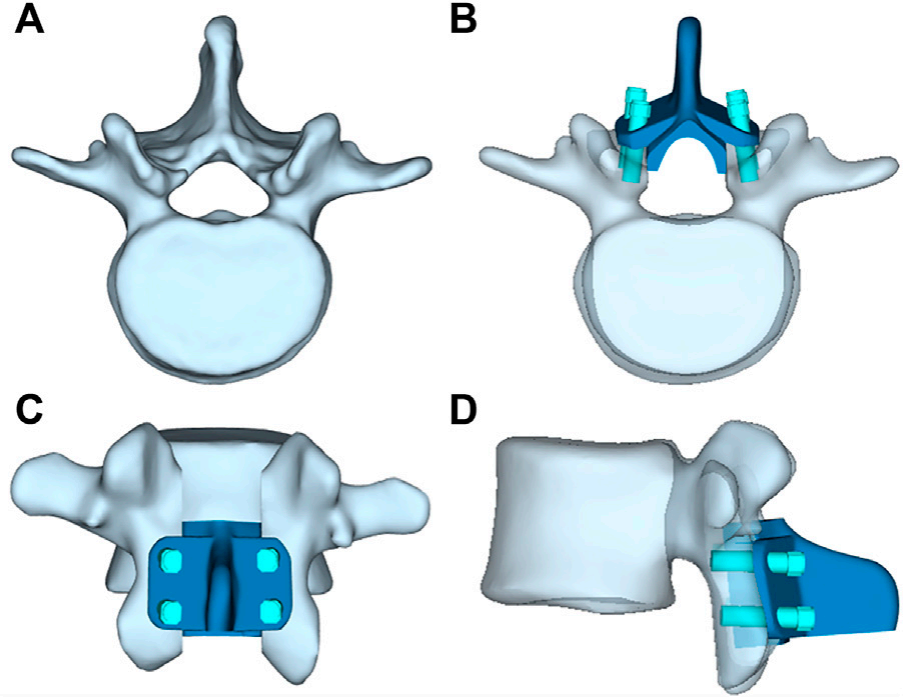
**(A)** L3 vertebrae, **(B)** Top view of artificil lamina, **(C)** Front view of artificil lamina,**(D)** Side view of artificil lamina.

The artificial lamina designed in this study is a bionic implant with a shape similar to the *in situ* vertebral plate to ensure successful decompression surgery. The L3 vertebral plate was removed during the laminectomy procedure, and the removed vertebral plate was trimmed, including thinning of the vertebral plate at the root of the spinous process to reduce mass, perforating the posterior spinous process to preserve the posterior ligament to reconstruct the posterior structure, and increasing the ventral curvature of the vertebral plate to expand the volume of the spinal canal so as to provide space for decompression. Finally, the modified lamina served as a template, and the lateral fixation structure was added to its posterior side, with the added lateral fixation structure conforming exactly to the posterior surface of the vertebral body. Holes were punched in the lateral lamina for screw fixation. The length and diameter of the screws were 20 mm and 4.5 mm, respectively. All the above-mentioned operations were performed on a 3-Matic 13.0 (Materialize, Leuven, Belgium). As a result, a complete model of the artificial lamina was obtained, as shown in Figure 2. The artificial lamina and fixation screws were made of Ti6Al4V.

### FE model of the surgical procedure

**FIGURE 3:**
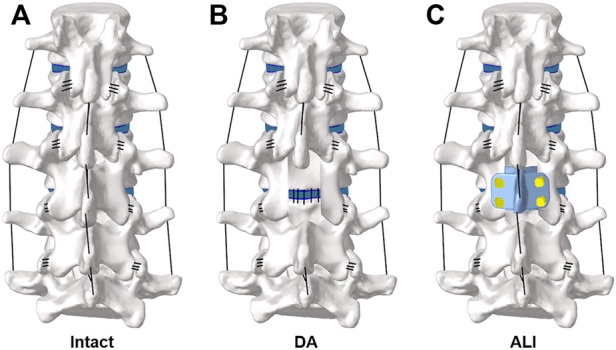
FE model of the surgical procedure: **(A)** Intact, **(B)** DA, **(C)** ALI.

A total of two surgical approaches were simulated and contrasted in this study, namely the lumbar decompression alone surgical model (DA) (Figure 3B) and the artificial lamina implantation surgical model (ALI) (Figure 3C). For the DA surgical model, it was simulated by removing the medial portion of the L3 vertebral body, the supraspinous ligament, the interspinous ligament, and the ligamentum flavum, while preserving the facet joints on both sides. For the ALI surgical model, artificial lamina was implanted in the laminectomy area and the supraspinous ligaments and interspinous ligaments were reconstructed. The material properties of the above devices are shown in Table 1.

### Boundary and loading conditions

For all FE models, the lower surface of the L5 vertebrae was constrained. the FE model loading process consisted of two steps. In the first step, a vertical axial preload of 500 N was applied to the upper surface of L1 as a representation of the upper body weight and muscle forces. In the second step, a 10 N-m moment was applied to the top center of the L1 vertebral body to simulate four movements of flexion, extension, lateral bending, and axial rotation for clinical prediction (Biswas et al., 2018; Song et al., 2021).

## Results

### Model validation

**FIGURE 4:**
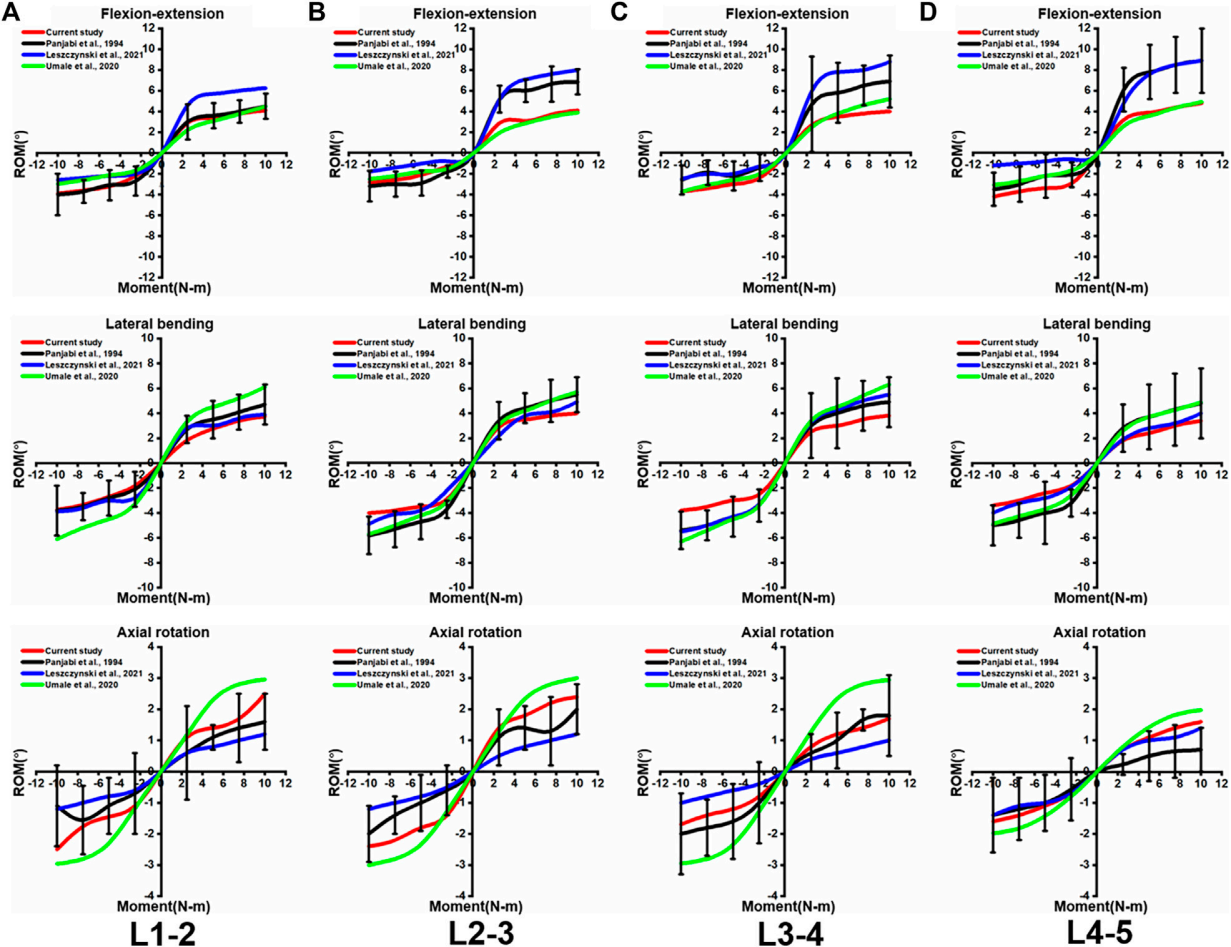
Comparison of the intersegmental ROM between the present intact model and the outcomes of previous publications under pure moments of 2.5N-m,5N-m,7.5n-m, and 10N-m conditions at **(A)** L1-2, **(B)** L2-3, **(C)** L3-4, and **(D)** L4-5. Error bars represent standard deviation.

**FIGURE 5:**
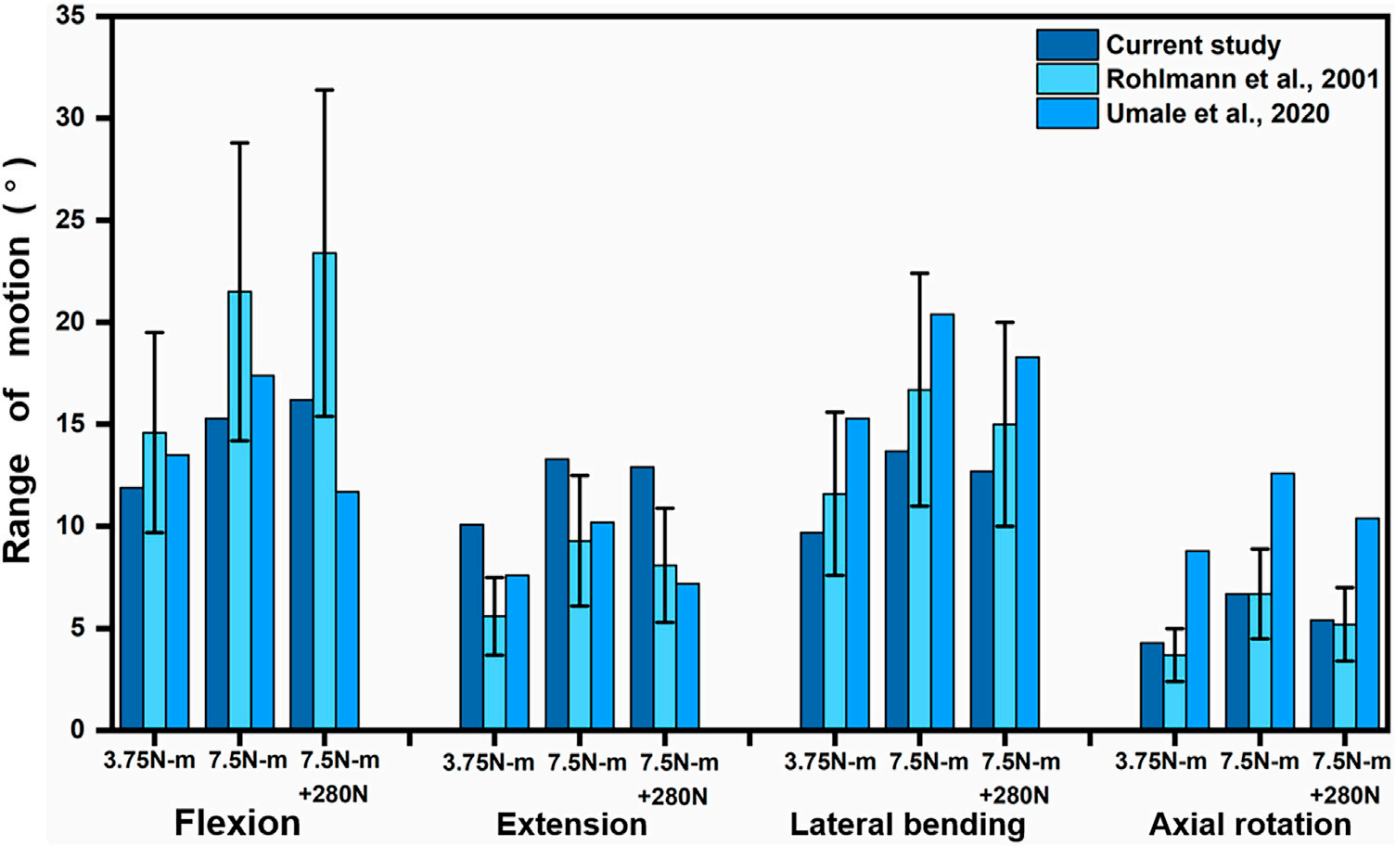
Comparison of L1-L5 total ROM between the present model and the results of previous publications under pure moments of 3.75N-m and 7.5N-m as well as 7.5N-m moments combined with 280N preload conditions at Flexion, extension, lateral bending, and axial rotation. Error bars represent standard deviation.

To validate the intact lumbar FE model, the intersegmental ROMs (L1/2, L2/3, L3/4, and L4/5) were compared with previous FE models and *in vitro* experimental results (Panjabi et al., 1994; Umale et al., 2020; Leszczynski et al., 2022) by applying incremental moments of up to 10 N-m at 2.5 N-m intervals to the upper surface of L1 for flexion, extension, lateral bending, and axial rotation tests (Figure 4). In addition, the total ROM was compared with previous data in the literature (Rohlmann et al., 2001; Umale et al., 2020) by applying pure moments of 3.75N-m,7.5N-m, and 7.5N-m moment combined with 280N preload on the upper surface of L1, respectively (Figure 5). After validation, the intersegmental ROM and total ROM of the intact lumbar FE model were consistent with previous literature, indicating that the L1-L5 intact FE model established in this study is valid and applicable for further clinical and experimental studies.

### Range of motion

The equations should be inserted in editable format from the equation editor. The ROM of all models in the L2/3, L3/4, and L4/5 segments are presented in Table 2. At the surgical segment (L3/4), the DA model showed the largest increase over the intact model in the flexion condition, followed by extension and axial rotation, which increased the least in lateral bending. The ROM increased by 52.5%, 10.3%, 2.5%, and 20.0% in the four conditions, respectively. In contrast, the ALI model had little effect on ROM, which increased only by 22.5% in flexion compared to the intact model, while remaining virtually unchanged in the other conditions.

**TABLE 2:** ROM of the surgical segment and adjacent segment (°).

		Flexion	Extension	Later bending	Axial rotation
Intact	L2/3	4.0	3.2	4.1	2.7
	L3/4	4.0	3.9	4.0	2.0
	L4/5	4.9	4.4	3.5	1.8
DA	L2/3	3.4	3.4	4.1	2.7
	L3/4	6.1	4.3	4.1	2.4
	L4/5	5.6	4.5	3.5	1.8
ALI	L2/3	4.3	2.9	4.1	2.8
	L3/4	4.9	3.7	4.0	2.0
	L4/5	5.3	4.4	3.5	1.9

At the adjacent segments (L2/3 and L4/5), no significant changes were observed in the DA model under lateral bending and axial rotation conditions. Whereas, the DA model increased by 6%–15% and 14.3%–23% in the cranial adjacent segment (L2/3) and caudal adjacent segment (L4/5) under lateral bending and axial rotation conditions, respectively. On the contrary, the ALI model showed a small change in ROM in the adjacent segment with a magnitude of less than 9.4%.

### Intradiscal pressure and maximum Von Mises stress of the annulus fibrosus

Table 3 shows the IDP and maximum Von Mises stress of the annulus fibrosus at the surgical and adjacent segments. The study indicated that the IDP and annulus fibrosus peak stress of the DA model increased at the surgical segment for all conditions. The increase was most pronounced in flexion, with 53.5% and 57.4% percent, respectively. Under other conditions, the IDP and annulus fibrosus stress peak increased by 0.4%–11.8% and 5.7%–8.9%, respectively. However, the ALI model increased the IDP and annulus fibrosis peak stress by 17.9% and 19.3% over the intact model only in flexion, while the change in all other conditions was less than 7.8%.

**TABLE 3:** Intradiscal pressure and maximum Von Mises stress of the annulus fibrosus (MPa).

		Flexion		Extension		Lateral bending		Axial rotation	
		IDP	Annulus fibrosus stress	IDP	Annulus fibrosus stress	IDP	Annulus fibrosus stress	IDP	Annulus fibrosus stress
Intact	L2/3	0.429	1.181	0.473	1.714	0.570	1.335	0.308	0.658
	L3/4	0.385	1.157	0.570	2.108	0.514	1.667	0.277	0.665
	L4/5	0.409	0.982	0.570	1.335	0.456	1.572	0.216	0.707
DA	L2/3	0.639	1.704	0.529	1.811	0.584	1.376	0.313	0.677
	L3/4	0.591	1.821	0.586	2.163	0.591	1.816	0.278	0.713
	L4/5	0.469	1.194	0.584	1.376	0.468	1.618	0.218	0.721
ALI	L2/3	0.476	1.257	0.440	1.580	0.571	1.334	0.307	0.655
	L3/4	0.454	1.380	0.563	2.088	0.512	1.651	0.274	0.651
	L4/5	0.440	1.082	0.571	1.334	0.456	1.571	0.216	0.706

The changes in the IDP and annulus fibrosus peak stress for the DA model at adjacent segments were similar to those of the surgical segments, i.e., the increase was most obvious in flexion, followed by extension, lateral bending, and axial rotation. In flexion, the IDP and annulus fibrosus peak stress increased by 46.7%–49% and 21.6%–44.3% at cranial adjacent segments and caudal adjacent segments, respectively.

Compared to the intact model, the ALI model showed no apparent changes in IDP and annulus fibrosus peak stress in all motion conditions, with changes ranging from 0.2% to 11% and 0.1–10.2%, respectively.

**FIGURE 6:**
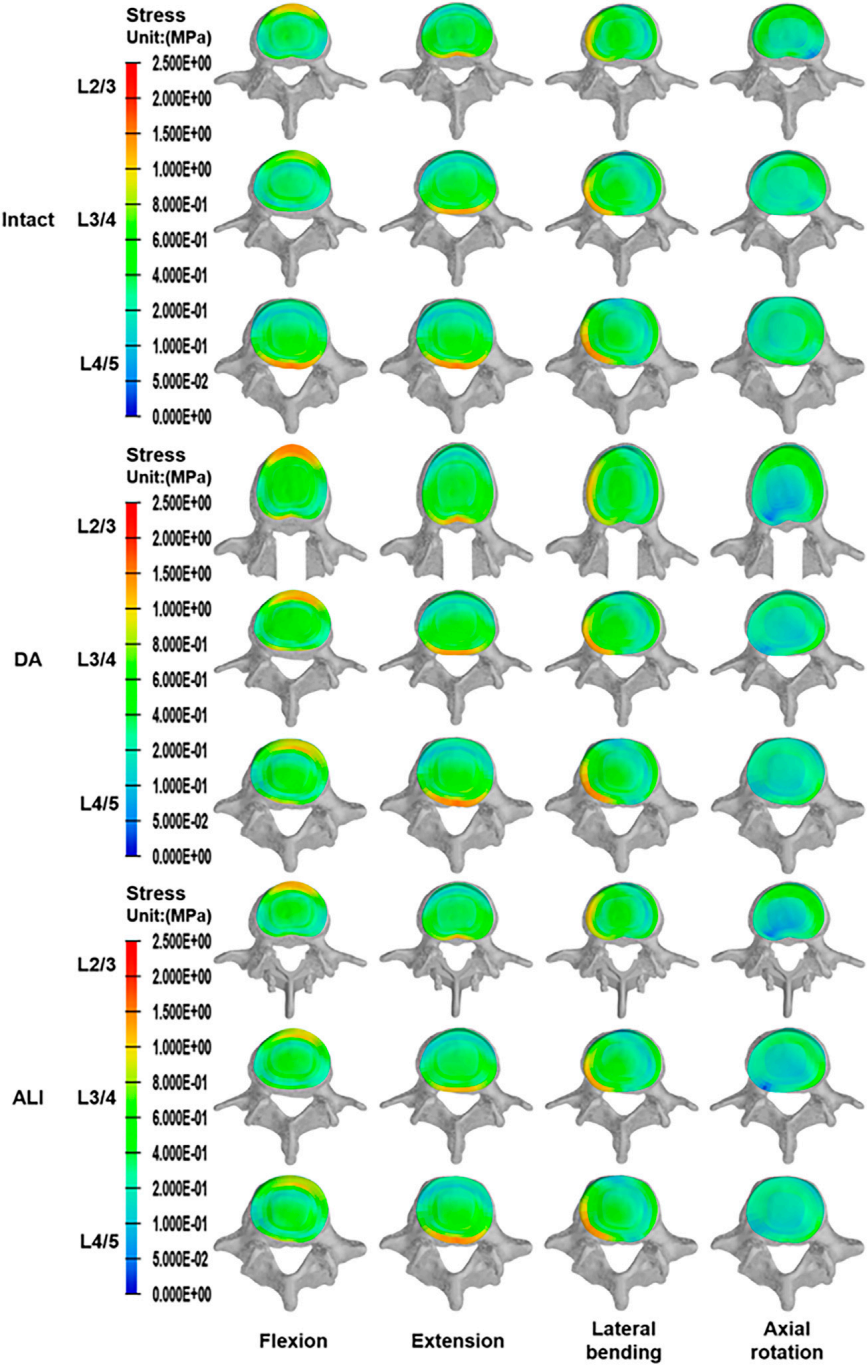
The distribution of disc stress at the surgical segment and adjacent segments.

As shown in Figure 6, the disc stresses are mainly concentrated on the edges of the disc under different motion conditions. In flexion and extension, the stresses were concentrated on the anterior and posterior sides of the disc. Similarly, in lateral bending, there was an obvious stress concentration on the compressed side of the disc and a tendency to decrease gradually toward the center of the disc. In axial rotation, the disc stress was concentrated on the lateral anterior side in the direction of axial rotation.

### Von Mises stress in the artificial lamina and screws

**FIGURE 7:**
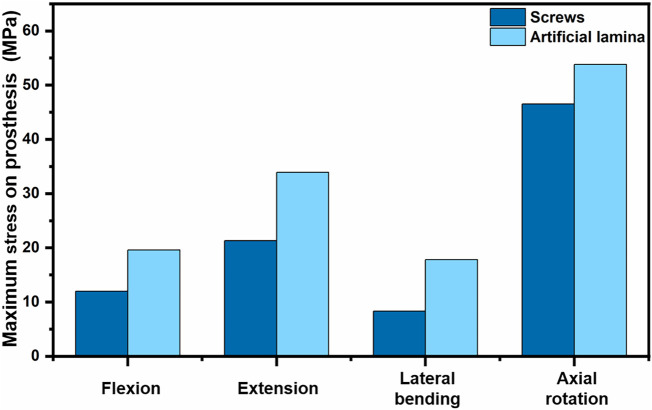
Stresses in the screws and artificial lamina.

**FIGURE 8:**
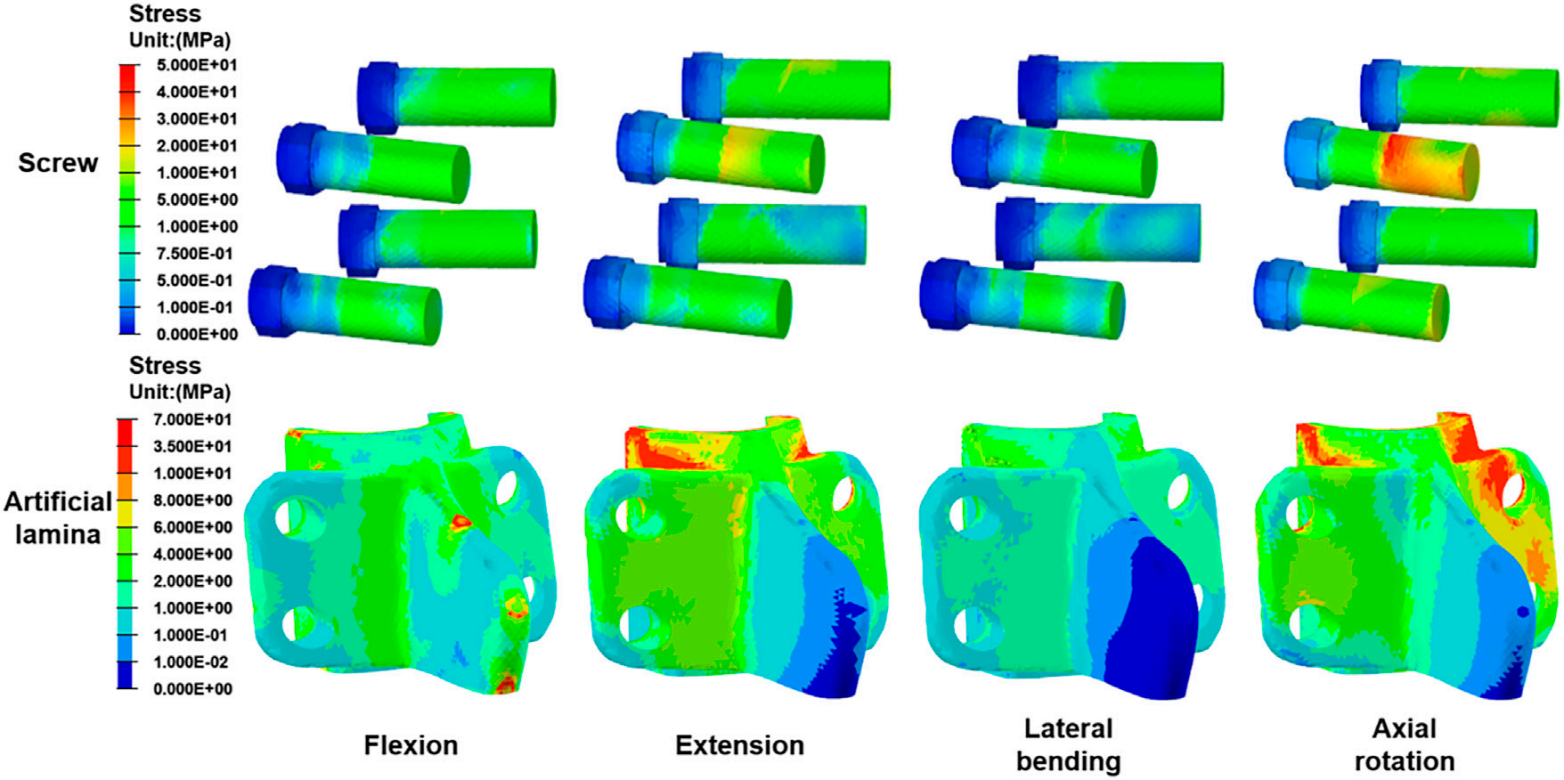
The stress distribution of screws and artificial lamina in all conditions.

Figure 7 shows the stress peak for screws and artificial laminas under different conditions. The peak stresses of the screws were 12.0 MPa, 21.3 MPa, 8.3 MPa, and 46.53 MPa in flexion, extension, lateral bending, and axial rotation, respectively, while the peak stresses of the artificial lamina were 19.6 MPa, 33.9 MPa, 17.8 MPa, and 53.84 MPa, respectively. In addition, the stress distributions of the screws and artificial lamina are shown in Figure 8. Under different conditions, the stresses of the fixed screws are mainly concentrated at the connection between the screws and the artificial lamina. The stresses in the artificial lamina were mainly concentrated at the root of the spinous process, around the screw hole, and at contact with the vertebral body. Moreover, there was an obvious stress concentration under the spinous process during flexion due to the stretching effect of the ligaments.

## Discussion

The artificial lamina can reconstruct the posterior ligamentous complex (PLC) after laminectomy to restore lumbar stability and provide a good mechanical barrier against epidural fibrous tissue proliferation, thereby reducing the incidence of FBSS (Lv et al., 2013; Li et al., 2020; Liu et al., 2021). However, a satisfactory match between the artificial lamina and the posterior spinal structure as well as the choice of artificial lamina material is crucial to restoring the function of the posterior spinal structure. Individualized reverse engineering methods are applied to build artificial lamina that can perfectly match posterior spinal structures with complex morphology. Titanium alloy material is the best substrate material for 3D printing of orthopedic implants because of its good mechanical strength and biocompatibility (Lv et al., 2015; Jing et al., 2020). Therefore, an individualized artificial titanium alloy lamina may be the best option to repair the defective area of the vertebral plate after laminectomy.

In this study, an individualized artificial titanium alloy lamina was designed based on lamina reimplantation and the ALI model was simulated like lamina reimplantation. Lamina reimplantation has previously been shown in studies to achieve good clinical results with reconstructed PLC (Li et al., 2019). A nonlinear inhomogeneous FE model of the whole lumbar spine was developed and simulated for both DA and ALI procedures to evaluate the biomechanical effects of the implanted artificial lamina. Briefly, the artificial lamina is implanted after laminectomy with preservation of the supraspinous and interspinous ligaments and then fixed with short screws to reduce the difficulty of the surgical procedure. At the same time, the supraspinous and interspinous ligaments are secured to the artificial lamina with silk threads. The lateral flanks of our artificial lamina fit closely to the residual bone surface, adequately filling the bone defect area and ensuring sufficient decompression space by increasing its ventral curvature. Then FEA was performed to measure the relevant indicators, including ROM, IDP, and annulus fibrosus peak stress at the surgical segment and adjacent segments.

The traditional laminectomy usually requires sacrificing the PLC to achieve the surgical purpose. However, PLC has a significant stabilizing effect on the spine and can restrict hyperflexion of the spine (Solomonow et al., 1998; Wu et al., 2018). As shown in our findings, the ROM of both the surgical and adjacent segments increased after laminectomy. In particular, the increase in ROM at the surgical level was most pronounced in flexion and axial rotation, with increases of 52.5% and 20%, respectively, which is consistent with the findings of Kim et al. (2015). Therefore, patients who were subjected to laminectomy should avoid hyperflexion and excessive axial rotation. Compared to the intact model, the ALI model increased the ROM of the surgical level in flexion by only 22.5%, and the rest of the conditions had a little change in ROM by 5%. In addition, compared with the DA model, the ROM at the surgical level of the ALI model was reduced by approximately 30% and 20% in flexion and axial rotation, respectively. It is because the ALI model restores the posterior tension band of the lumbar spine and reconstructs the PLC structure, in which the lamina is resistant to axial rotation and the posterior tension band is resistant to flexion. The study showed that preserving the posterior tension band structure is beneficial for improving lumbar symptoms, reducing the incidence of complications, and providing early recovery of function (Xue et al., 2022). Interestingly, Gillespie and Dickey, (2004) found through their study that the contribution of the ligamentum flavum to resist flexion was 24.7%. In the present study, the ROM of the ALI model in flexion was still increased after PLC reconstruction, which might be attributed to ligamentum flavum failure to reconstruct. However, it was generally found that the ALI model had less effect on ROM, which highlights the ability of the artificial lamina to not only reconstruct the stability of the spine but also to preserve the original ROM of the spine.

In this study, the IDP and the annulus fibrosus stresses were increased in both the surgical segment and the adjacent segment after laminectomy, especially in flexion. Compared with the intact model, the IDP and annulus fibrosus stresses at the surgical segment and the adjacent segments increased by 21.6%–53.5% in the DA model in flexion, while only 6.4%–19.3% in the ALI model. The IDP and annulus fibrosus stresses were also slightly greater in the DA model than in the ALI model under other conditions. This may be due to the fact that when the PLC is damaged, the posterior spinal structures lose their resistance to compressive forces and most of the compressive forces are transferred to the intervertebral disc (Merter et al., 2019). A spinous osteotomy study showed that preserving the PLC can reduce disc stresses in the decompressed segment (Li et al., 2017). Huang et al. (2016) found that preservation of the PLC in posterior lumbar interbody fusion surgery was effective in preventing the incidence of adjacent segmental disease (ASD). Furthermore, another study also found that laminectomy alone also caused ASD (Bydon et al., 2016). As shown in our results, increased IDP and annulus fibrosus stress at adjacent segments after laminectomy probably resulted in it. Therefore, we could deduce that artificial lamina is helpful to prevent ASD and reduce disc stress in the decompressed segment by restoring posterior spinal structures. The ligamentum flavum can create a resistance effect in flexion to reduce stresses in the anterior part of the disc (Merter et al., 2019), so the IDP and annulus fibrosus stresses of the ALI model are increased in flexion. Our results also confirm that the posterior spinal structures have a greater influence on disc stress values and a smaller influence on disc stress distribution, which is consistent with the description by Zander et al. (2003).

The stress distribution of the artificial lamina is gradually dispersed along with the spinal roots. The maximum stress occurs in axial rotation and is mainly concentrated on the contact surface between the artificial lamina and the vertebral body. The stresses on the fixation screws were also greatest in axial rotation and were mainly concentrated on the contact between the screws and the artificial lamina. This suggests that the lamina produces greater resistance stresses in axial rotation, which is consistent with the findings reported by Liu et al. (2021). Besides, a titanium plate fixation with lamina reimplantation found that restoration of the posterior spinal structures maintained resistance to axial rotation and maintained spinal stability (Nong et al., 2015). Another interesting phenomenon is that a significant stress concentration occurs in flexion at the artificial lamina-ligamentous junction, while not in other conditions, which is determined by the characteristic of the ligament being subjected to only tension and not pressure. Therefore, excessive flexion should be avoided after the implantation of the artificial lamina.

In conclusion, the artificial lamina can decrease ROM, IDP, and annulus fibrosus stresses at the surgical segment and adjacent segments by reconstructing PLC, which demonstrates biomechanically the role of artificial titanium lamina in stabilizing the lumbar spine and reducing ASD.

There are still some limitations in this study. In the study of Elmasry et al. (2017); Elmasry et al. (2018), the intervertebral disc was defined as poroelastic material, consisting of a solid phase embedded in a fluid media, which is more in line with the real disc material properties, whereas in the present experiments the disc was only defined as a simple hyperelastic material, so the modeling approach needs further refinement. Since the elastic modulus of titanium alloy is 9.2 times higher than cortical bone, the stress shielding phenomenon and stress concentration are inevitable. Topology optimization techniques can effectively decrease the modulus of elasticity of the prosthesis to avoid stress shielding (Bartolomeu et al., 2020). Therefore, the manufacture of topologically optimized individualized artificial lamina for clinical treatment is a better choice. In addition, the experiment still needs further animal or in vitro-related experiments to validate.

## Conclusion

Compared with the DA model, the ALI model reduces ROM, IDP, and annulus fibrosus stresses at the surgical segment and adjacent segments. The ALI model better preserves the biomechanical properties of the intact lumbar spine. It is biomechanically demonstrated that the artificial lamina has the potential advantage of reducing the risk of ASD. However, there is still a degree of hypermobility and increased disc stress in flexion after artificial lamina implantation, so patients undergoing artificial lamina implantation should avoid excessive flexion that could cause secondary injury. These findings are expected to provide a theoretical basis for the further clinical application of the artificial lamina.

## Data Availability

The original contributions presented in the study are included in the article/supplementary material, further inquiries can be directed to the corresponding authors.
